# Fetal MRI-Based Mediastinal Shift Angle (MSA) and Percentage Area of Left Ventricle (pALV) as Prognostic Parameters for Congenital Diaphragmatic Hernia

**DOI:** 10.3390/jcm13010268

**Published:** 2024-01-03

**Authors:** Greta Thater, Lara Angermann, Silviu-Viorel Virlan, Christel Weiss, Neysan Rafat, Michael Boettcher, Julia Elrod, Tom Bayer, Oliver Nowak, Stefan O. Schönberg, Meike Weis

**Affiliations:** 1 Department of Radiology and Nuclear Medicine, University Medical Center Mannheim, Theodor-Kutzer-Ufer 1-3, 68167 Mannheim, Germany; greta.thater@umm.de (G.T.); silviu-viorel.virlan@umm.de (S.-V.V.);; 2 Department of Medical Statistics, Biomathematics and Information Processing, University Medical Center Mannheim, Theodor-Kutzer-Ufer 1-3, 68167 Mannheim, Germany; christel.weiss@medma.uni-heidelberg.de; 3 Department of Neonatology and Neonatal Intensive Care Medicine, Hospital Stuttgart, Kriegsbergstraße 60, 70174 Stuttgart, Germany; n.rafat@klinikum-stuttgart.de; 4 Department of Pediatric Surgery, University Medical Center Mannheim, Theodor-Kutzer-Ufer 1-3, 68167 Mannheim, Germany; michael.boettcher@umm.de (M.B.); julia.elrod@umm.de (J.E.); 5 Department of Neonatology, University Medical Center Mannheim, Theodor-Kutzer-Ufer 1-3, 68167 Mannheim, Germany; tom.bayer@umm.de; 6 Department of Obstetrics and Gynecology, University Medical Center Mannheim, Theodor-Kutzer-Ufer 1-3, 68167 Mannheim, Germany; oliver.nowak@umm.de

**Keywords:** congenital diaphragmatic hernia (CDH), mediastinal shift angle (MSA), percentage area of left ventricle (pALV), prenatal diagnosis of CDH, prognostic parameters CDH, fetal MRI, ECMO, CLD, obstetrics and gynecology

## Abstract

Objective: Fetal magnetic resonance imaging (MRI) is broadly used as a method for assessing prognosis in congenital diaphragmatic hernia (CDH). In addition to the extent of lung hypoplasia, determined by measuring the lung volume, cardiac impairment due to pulmonary hypertension and left cardiac hypoplasia is decisive for the prognosis. The percentage area of left ventricle (pALV) describes the percentage of the inner area of the left ventricle in relation to the total area, whereas the mediastinal shift angle (MSA) quantifies the extent of cardiac displacement. The prognostic value of pALV and MSA should be evaluated in terms of survival, the need for extracorporeal membrane oxygenation (ECMO) therapy, and the development of chronic lung disease (CLD). Methods: In a total of 122 fetal MRIs, the MSA and pALV were measured retrospectively and complete outcome parameters were determined regarding survival for all 122 subjects, regarding ECMO therapy in 109 cases and about the development of CLD in 78 cases. The prognostic value regarding the endpoints was evaluated using logistic regression and ROC analysis. Results: The MSA was significantly higher in children who received ECMO therapy (*p* = 0.0054), as well as in children who developed CLD (*p* = 0.0018). ROC analysis showed an AUC of 0.68 for ECMO requirement and 0.77 with respect to CLD development. The pALV showed a tendency towards higher levels in children who received ECMO therapy (*p* = 0.0824). The MSA and the pALV had no significant effect on survival (MSA: *p* = 0.4293, AUC = 0.56; pALV: *p* = 0.1134, AUC = 0.57). Conclusions: The MSA determined in fetal MRI is a suitable prognostic parameter for ECMO requirement and CLD development in CDH patients and can possibly be used as a supplement to the established parameters.

## 1. Introduction

Congenital diaphragmatic hernia (CDH) is a rare disease with a prevalence of 1-2:3000 newborns [1,2,3]. It is manifested by a congenital defect of the diaphragm, leading to the herniation of abdominal organs into the thoracic cavity. This anomaly leads to pulmonary hypoplasia, which, depending on its severity, can result in significant pulmonary and cardiovascular impairment as well as postnatal complications. In cases of marked pulmonary hypoplasia, adaptive pulmonary hypertension occurs to maintain lung perfusion. This in turn leads to right heart hypertrophy and consecutive left heart hypoplasia.

Failure to diagnose CDH prenatally can lead to inappropriate postnatal care, worsening pulmonary hypertension and significantly reducing postnatal survival. Therefore, early interdisciplinary antenatal care in specialized care centers proves to be crucial for postnatal outcomes. The University Hospital of Mannheim is a high-frequency, tertiary care center and cares for more than 60 newborns with CDH every year. The initial examination is performed by sonography in the obstetrics department, followed by fetal magnetic resonance imaging (MRI) for further evaluation. Prenatal imaging for the diagnosis and prognosis of CDH is subject to constant progress, and the use of fetal magnetic resonance imaging has become increasingly important. The non-invasive technique and high spatial resolution of fetal MRI allow precise morphological analysis of affected fetuses. In fetal MRI of CDH patients, the observed-to-expected FLV (o/e FLV) according to the formula of Rypens et al. has been shown to be a reliable prognostic parameter for several endpoints such as the postnatal need for extracorporeal membrane oxygenation therapy (ECMO) and survival [4].

Because of the significant dependence of the course of isolated congenital diaphragmatic hernia (CDH) on the extent of lung hypoplasia, prenatal counselling is guided primarily by prenatal estimation of lung volume. Diverse prognostic parameters have been designed for both ultrasound and magnetic resonance imaging (MRI), among which the observed-to-expected lung head ratio (o/e LHR) and the MRI-based observed-to-expected fetal lung volume ratio (o/e FLV) stand out as essential. The randomized TOTAL (Tracheal Occlusion to Accelerate Lung Growth) trial by Deprest et al. already focuses on the significant importance of o/e LHR on ultrasound as it serves as the basis for decision-making regarding therapeutic fetal tracheal occlusion (FETO) intervention. This interventional measure has been associated with a remarkable survival benefit in severe cases of left diaphragmatic hernia [5,6].

In addition to the extent of pulmonary hypoplasia, cardiac impairment due to pulmonary hypertension and left cardiac hypoplasia is decisive for prognosis. Siebert et al. (1984) already classified left heart hypoplasia as a relevant risk factor in the pathogenesis of heart failure in patients with left-sided CDH [7]. Recent studies by Patel et al. (2019) and Prasad et al. (2022) came to the same conclusion [8,9]. The percentage area of the left ventricle to the total area of the ventricles (pALV) could serve as a quantitative parameter for this, which indicates cardiac stress and enables a differentiated assessment of the severity of CDH and potential cardiovascular consequences. 

The mediastinal shift angle (MSA) can be determined by MRI and quantifies the extent of cardiac displacement. Previous studies have looked at the MSA in relation to left-sided hernias and have predominantly investigated its value as an MRI prognostic parameter in direct comparison with already established parameters such as the o/e LHR and o/e TFLV [10,11,12]. Only Wang et al. (2022) and Savelli et al. (2020) address direct outcome parameters such as survival and mortality, respectively, and respiratory-cardiovascular prognosis [12,13]. All studies ignored the rarer and smaller, but more frequently severely affected, subpopulation of right-sided hernias.

The aim of the present study is to evaluate the prognostic value and significance of the new MRI prognostic parameters pALV and MSA with regard to survival, the need for extracorporeal membrane oxygenation (ECMO) therapy, and development of chronic lung disease (CLD) both in relation to the overall collective of left and right diaphragmatic hernias and in relation to the subpopulations of left- and right-sided hernias in a large data collective (*n* = 122).

## 2. Materials and Methods

### 2.1. Patient Cohort 

All fetal MRI scans between 2016 and 2021 have been retrospectively analyzed. All MRI scans have been performed on a 1.5 Tesla MRI system (Magnetom Avanto, Siemens Healthineers, Forchheim, Germany) as previously described [14]. Exclusion criteria were additional malformations, incomplete examination protocols, and MRI images of insufficient assessability due to fetal movement artifacts. In cases of multiple MRI scans, only the last MRI scan was considered. This retrospective analysis was approved by the local research ethics committee (approval number: 2021-667). 

A total of 206 children were examined. Patients who only had external fetal MRI imaging (*n* = 22) were excluded for quality assurance and to ensure uniform examination standards. A lack of evaluability of existing sequences (*n* = 50), for example, due to movement artifacts of the fetus, lack of the four-chamber view, or incorrect sectional plane led to exclusion from the study. In addition, an incomplete examination protocol with missing individual MRI sequences (*n* = 11) and therefore an incomplete collection of MRI parameters (pALV, MSA, o/e FLV, PPLV, mediastinal and thoracic volume) also led to exclusion from the study. The presence of other malformations such as CPAM (*n* = 1) also led to exclusion from the study. In the end, 122 fetal MRI scans of fetuses diagnosed with congenital diaphragmatic hernia (CDH) could be included in the analysis. Within this cohort, 111 fetuses (91%) had left-sided CDH and 11 fetuses (9%) had right-sided CDH. The included fetuses were on average at a developmental stage of 30 + 5 weeks of gestation (mean: 30.73). Complementary prenatal and postnatal detailed information on all 122 patients can be found in the Supplementary Material.

### 2.2. Outcome Parameter

This study investigated the prognostic value of the MRI parameters pALV and MSA concerning survival, the need for ECMO, and the development of CLD. 

The criteria for the use of ECMO therapy in CDH patients were derived from the consensus paper by the CDH EURO Consortium (2015) [15]. CLD was defined inconsistently for a long time. In the present study, CLD was defined by the persistent oxygen demand after 28 days of life. 

The data entered in the clinical information system (CIS) formed the basis for recording the outcome parameters. The recording of complete outcome parameters was carried out accordingly depending on the available clinical information. In the process, data regarding survival could be determined in all 122 cases, the need for extracorporeal membrane oxygenation therapy (ECMO therapy) was recorded in 109 cases, and the development of chronic lung disease (CLD) was documented in 78 cases. 

More precisely, the case numbers were broken down as follows: The need for ECMO was known for all 122 patients. A total of 45 patients required ECMO therapy and 64 patients did not require ECMO. A total of 13 patients did not receive ECMO due to contraindications for ECMO, because their condition was too poor for ECMO and there was no realistic likelihood of survival or the parents and physicians decided against a life-altering ECMO attempt. These 13 patients were not counted as “no ECMO” but were excluded from the statistical analysis of outcome parameters. This results in statistically usable data for the ECMO parameter from 109 children. 

The data were considered evaluable if the patient was known to be in hospital until the 28th day of life. Accordingly, out of the 122 patients who were examined, 30 were diagnosed with CLD and 48 did not have CLD. Patients who were no longer in hospital on day 28, for example, because they were discharged or transferred to hospitals close to home in a stable condition with oxygen, were excluded from the statistical analysis. This totaled 19 patients. A total of 25 patients died in hospital before the 28th day of life. Accordingly, no CLD could be collected for these children on the 28th day of life. 

### 2.3. MSA Measurement

The mediastinal shift angle (MSA) was calculated in a total of 122 fetal MRI examinations by one investigator with one year of experience in MRI imaging under the supervision of an experienced investigator with 10 years of experience. Image viewing, processing, and segmentation were performed using the reporting and post-processing software aycan workstation (aycan Digitalsysteme GmbH, Würzburg, Germany).

**Figure 1:**
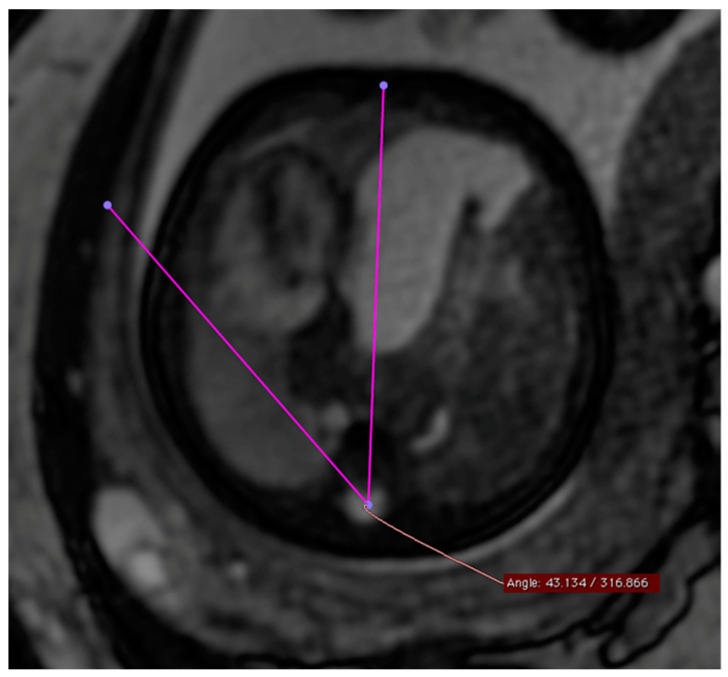
Measurement of MSA (mediastinal shift angle).

The MSA was quantified using a T2-weighted steady-state sequence (“True fast imaging with steady-state free-precision”—TRUFI) in the transverse section of the fetus in the four-chamber view. The selection of the TRUFI sequence resulted from its ability to clearly delineate the heart from the adjacent lung parenchyma. The use of the four-chamber view as a reference plane ensured easy reproducibility of the angle measurement (Figure 1). To perform the actual angle measurement, a straight line was drawn from the center of the sternum to the center of the spinal canal of the thoracic vertebral body of the same height. A second straight line was drawn from the spinal canal center of the corresponding thoracic vertebral body as a tangent at the outermost point of the pericardial side facing the diaphragmatic defect. The resulting smaller angle α between these two straight lines corresponded to the MSA.

This figure illustrates a T2 steady-state free-precision (TRUFI) sequence in the axial section plane (uniform four-chamber plane) of a fetal MRI scan in a patient with left congenital diaphragmatic hernia (CDH). In this MRI representation, the measurement of the mediastinal shift angle α is visualized as an example.

### 2.4. Percentage Area of Left Ventricle (pALV)

The calculation of the left ventricular area percentage of the total ventricular area or “percentage Area of Left Ventricle” (pALV) was performed for a total of 122 fetal MRI examinations. Image viewing, processing, and segmentation were performed using the reporting and post-processing software aycan workstation (aycan Digitalsysteme GmbH, Würzburg, Deutschland). The measurements were always performed by one and the same person, a doctor in training, who was trained in advance in both measurement and viewing of fetal MRI images and was accompanied during the segmentation process by another doctor in training. The measurements were validated by another person, a senior physician. 

**Figure 2:**
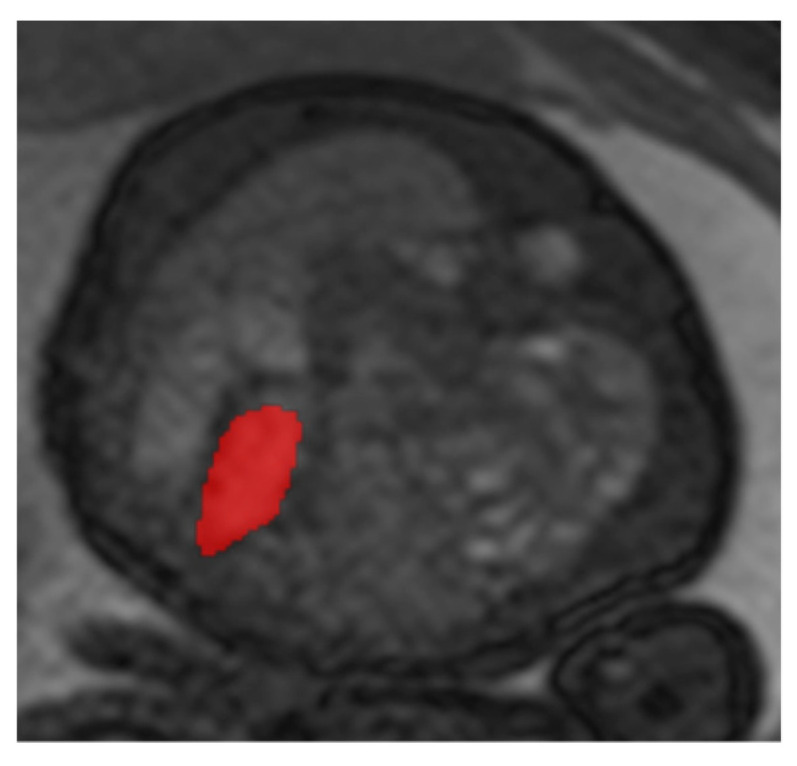
Measurement of pALV (percentage area of left ventricle).

The measurement procedure was analogous to the determination of the mediastinal shift angle (MSA) using T2-weighted sequences. For the determination of the cardiac parameter “percentage Area of Left Ventricle” (pALV), which represents the percentage area of the left ventricle, the absolute areas of both inner ventricular surfaces of the heart are first measured in their greatest extent in the four-chamber view on a transverse sectional plane in relation to the fetus (Figure 2). For this purpose, the inner surfaces of the right and left ventricles were segmented, and the surface was automatically calculated by postprocessing.

This figure illustrates a T2 steady-state free-precision (TRUFI) sequence in the axial slice plane (uniform four-chamber plane) of a fetal MRI scan in a patient with left congenital diaphragmatic hernia (CDH). This MRI representation shows an example of the segmentation of the right inner ventricular surface (red surface in the figure).

Subsequently, the absolute values of the inner ventricular areas were calculated according to the following formula in order to obtain the percentage of the area of the left ventricle:

$$pALV\left(\%\right)=\frac{ALV}{ALV+ARV}$$

*ALV* (Area of Left Ventricle) = absolute value of the inner ventricular surface of the left ventricle in cm^2^.

*ARV* (Area of Right Ventricle) = absolute value of the inner ventricular surface of the right ventricle in cm^2^.

### 2.5. Statistical Analysis

We investigated whether the outcome parameters (Survival—Non-Survival; ECMO—No ECMO and CLD—No CLD) differed significantly for the MRI parameters MSA and pALV via a *t*-test. If the requirement of variance homogeneity was not met, the Welch test was used instead. 

Binary logistic regression and ROC analysis were used to evaluate the prognostic value of the MRI parameters MSA and pALV with respect to the endpoints (survival, ECMO therapy, and CLD). The statistical analysis was performed using dedicated analytic software (SAS Analytics Software, version 9.4 (TS1M8). The graphical representation of the logistic regressions by ROC curves was done with the help of a spreadsheet program (Microsoft Excel^®^ 2016, version 16.0.5422.1000).

## 3. Results

### 3.1. Prognostic Value of the MSA

#### 3.1.1. Survival/Non-Survival

**Figure 3:**
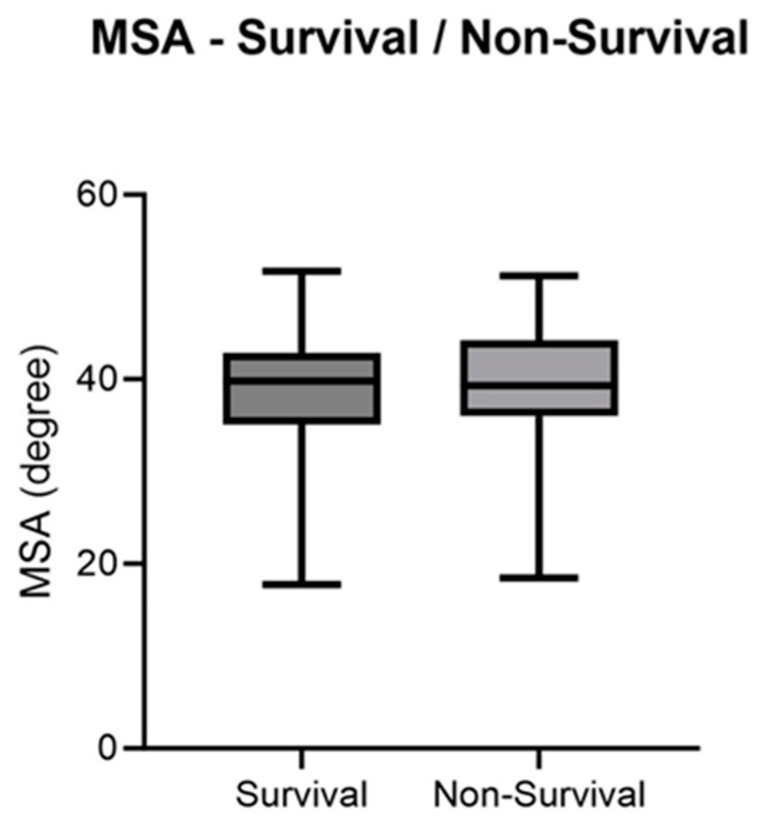
MSA—Survival/Non-Survival.

For patients with congenital diaphragmatic hernia who survived, the mean MSA was 38.58°. The mean MSA of patients with CDH who died was 39.59°. The distribution of values is visualized in Figure 3. 

This graph shows the value distribution of non-survival and survival based on the MSA as boxplots with minimum and maximum values, standard deviation, and mean value (*p* = 0.1134).

For the included cohort of CDH patients, there is no significant relationship between MSA and the survival of the patients (*p* = 0.4293, Table A1). In line with this, the AUC value was 0.563. 

In addition, for the left- and right-sided hernia subgroups, there is no significant prognostic influence of the MSA on the survival of the patients (*p* = 0.5043; *p* = 0.5537). The right-sided hernia subgroup had a slightly higher AUC value (0.625) than the overall collective and the left-sided hernia subgroup.

#### 3.1.2. ECMO

**Figure 4:**
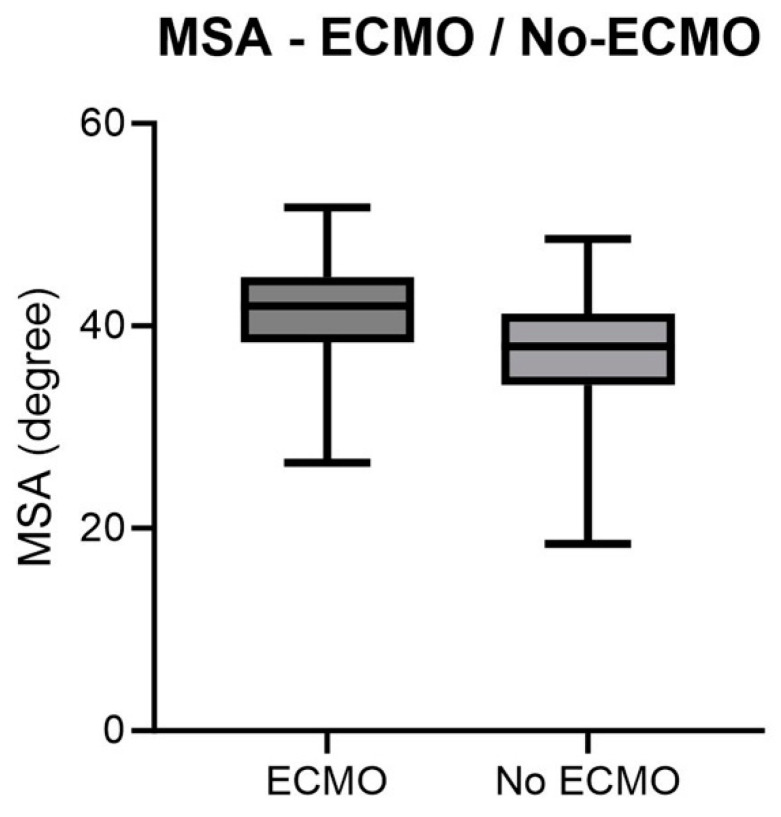
MSA—ECMO.

The MSA was significantly higher in patients receiving ECMO therapy (*p* = 0.0035). On average, the MSA in patients who received ECMO therapy was 40.95° with a mean ECMO duration of 9.88 days (Figure 4). The prognostic predictive power of the MSA was significant for patients who received ECMO (*p* = 0.0054, AUC = 0.678). 

This graph lists the value distribution of ECMO demand based on the MSA as boxplots with minimum and maximum values, standard deviation, and mean (*p* = 0.0054).

The results of the analysis of the mediastinal angle as a prognostic parameter for ECMO therapy of both the total collective and the subgroupings of right-sided hernias and left-sided hernias are shown in Table A1. 

**Figure 5:**
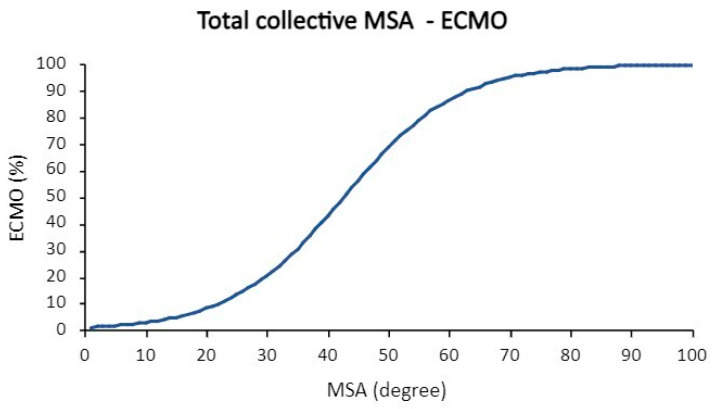
Logistic regression MSA-ECMO in the total collective.

A fetus with an MSA of 30° would have a 21% probability of receiving ECMO therapy. With an MSA of 50°, ECMO therapy would be necessary with a probability of 69% (see Figure 5). 

The logistic regression demonstrates that the ECMO requirement probability depends on the MSA. The mathematical formula for the corresponding logistic regression can be found in Appendix A (Formula (A1)). ROC analysis revealed an AUC of 0.68.

For the right-sided hernia subgroup receiving ECMO therapy, the MSA was not significantly higher. The AUC of MSA regarding ECMO in right-sided hernias was 0.938. Applying the calculation example using the appropriate logistic regression formula shows that a fetus with a right-sided hernia and an MSA of 36° requires ECMO therapy in only 4% of cases, while there is a 96% probability of no need for ECMO therapy. In contrast, an MSA of 50° leads to a need for ECMO therapy with almost 100% certainty.

#### 3.1.3. CLD

**Figure 6:**
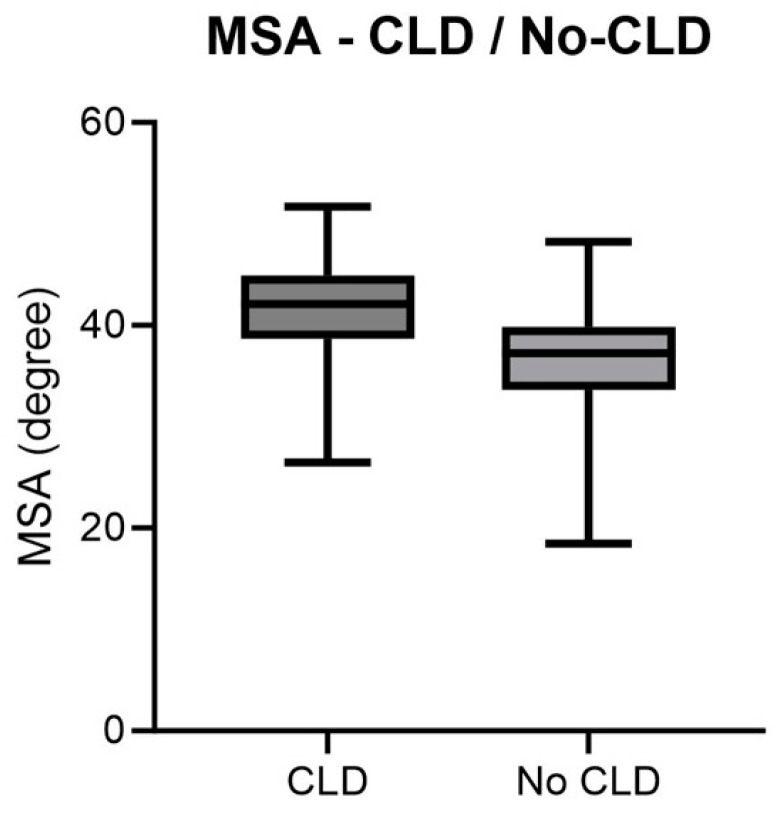
MSA—CLD.

Patients with congenital diaphragmatic hernia who developed CLD had a mean MSA of 41.14° and patients with a mean MSA of 36.35° did not develop CLD. Consequently, the MSA was significantly higher for patients with CLD (*p* = 0.0004). The distribution of values is visualized in Figure 6. 

This graph lists the value distribution of CLD development based on the MSA as boxplots with minimum and maximum values, standard deviation, and mean (*p* = 0.0018). 

The results of the analysis of the mediastinal angle as a prognostic parameter for CLD of both the total collective and the subgroupings of right-sided and left-sided hernias are shown in Table A1.

When considering the total population, the MSA was significant in patients with CLD (*p* = 0.0018) with an AUC of 0.766.

**Figure 7:**
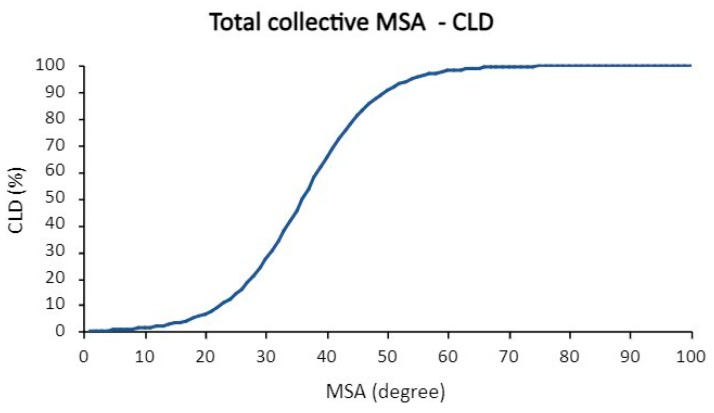
Logistic regression MSA-CLD of the total collective.

Thus, a fetus with CDH and an MSA of 30° develops CLD at a rate of 27%. If, on the other hand, the MSA is 50°, CLD would be 91% likely (Figure 7). 

The logistic regression demonstrates that the CLD development probability is dependent on the MSA. The mathematical formula for the corresponding logistic regression can be found in Appendix A (Formula (A2)). ROC analysis revealed an AUC of 0.77.

Within the subpopulation of right-sided hernias, no statistically significant correlation between the MSA and the development of CLD could be proven (*p* = 0.3422). ROC analysis resulted in an AUC of 0.929 with regard to CLD development in the right-sided hernia subgroup. For example, a fetus with right-sided CDH and an MSA of 35° has a 1% probability of developing CLD. In contrast, an MSA of 50° results in an almost 100% probability of developing CLD.

### 3.2. Prognostic Value of the pALV

#### 3.2.1. Survival/Non-Survival

The results of the analysis of the pALV as a prognostic parameter for the survival of both the total collective and the subgroupings of right- and left-sided hernias are summarized in Table A2. 

For patients with congenital diaphragmatic hernia who survived, the mean pALV was 49.53%. The mean pALV of patients who died was 48.08%. There was no significant correlation between pALV and survival (*p* = 0.111). 

The pALV did not exert a significant influence on survival in the CDH cohort (*p* = 0.1134). This is supported by the analysis of the ROC curve, which yielded an AUC value of 0.570. A significant association between pALV and survival was neither found in the total collective nor for the subgroups of right-sided hernias and left-sided hernias.

#### 3.2.2. ECMO

Patients who received ECMO therapy had an average pALV of 48.19% and required ECMO therapy for 9.88 days. CDH patients without ECMO had an average pALV of 49.70% (*p* = 0.079).

The results of the analysis of the pALV as a prognostic parameter for the need for ECMO therapy of both the total collective and the subgroupings of right- and left-sided hernias are summarised in Table A2. ROC analysis showed an AUC of 0.597 with respect to ECMO requirement, so there was no significant correlation between the pALV and the need for ECMO therapy for the overall population (*p* = 0.0824). 

For both right-sided and left-sided hernias, the pALV does not significantly influence the need for ECMO therapy. 

#### 3.2.3. CLD

The results of the analysis of the pALV as a prognostic parameter for the development of CLD of both the total collective and the subgroupings of right- and left-sided hernias are summarised in Table A2. 

Patients with CLD had a mean pALV of 48.64%. For patients who did not develop CLD, the mean pALV was 49.79%. There was no significant correlation between the pALV and the development of CLD (*p* = 0.219). 

The results of the logistic regression analysis show that the pALV does not significantly influence the occurrence of CLD (*p* = 0.2173, AUC = 0.588). The analysis was carried out for the subgroups of right- and left-sided hernias in line with the overall population. 

## 4. Discussion

The present study evaluates the prognostic value and significance of the new MRI prognostic parameters pALV and MSA regarding various clinical endpoints: survival, need for extracorporeal membrane oxygenation (ECMO) therapy, and development of chronic lung disease (CLD), both in relation to the overall collective of left- and right-sided diaphragmatic hernias and in relation to the subpopulations of left- and right-sided hernias. The mediastinal displacement angle (MSA) can be determined using MRI and quantifies the extent of cardiac displacement. Previous studies have considered the MSA regarding solely left-sided hernias and investigated its prognostic value in direct comparison with already established parameters such as the o/e LHR and o/e TFLV [10,11,12]. 

According to Amodeo et al. (2022), the MSA and o/e TFLV correlate inversely, *p* < 0.001 [10]. In their analysis of left-sided hernias, Ding et al. (2023) even tested the MSA value as being analogous to established prognostic parameters such as o/e LHR [11]. He also showed that increased MSA was significantly associated with mechanical ventilation and prolonged pharmacotherapy and relevantly contributed to prolonged hospital stay [10].

Only Wang et al. (2022) and Savelli et al. (2020) addressed direct outcome parameters such as survival or mortality and respiratory/cardiovascular prognosis [12,13]. According to Savelli et al. (2020), the AUC for the predictive power of survival was 0.931 [13]. Our analysis showed that the MSA had no significant effect on the survival of CDH patients. While the o/e FLV is already established as a prognosis parameter for survival estimation, the MSA appears to be an insufficient indicator for survival in our study cohort.

Previous studies have not considered the relationship between increased MSA and the need for ECMO therapy or the development of CLD as important cardiocirculatory and pulmonary complications. In particular, the need for ECMO therapy may have a relevant impact on the length of hospital stays, which has already been investigated in the study by Amodeo et al. (2022) and is becoming increasingly important [10]. 

A significantly higher in MSA was demonstrated for the overall population in correlation with ECMO therapy (*p* = 0.0035). The AUC value of around 0.7 implies a certain predictive capacity of the MSA regarding the need for ECMO therapy. In the context of right-sided hernias, no significant MSA was observed for those requiring ECMO therapy (*p* = 0.3380). Nevertheless, the MSA with an AUC value of more than 0.9 for right-sided hernias might indicate its value, and further investigations with a higher number of patients must be performed. 

Within the overall population, there was a significant association between increased MSA and the development of chronic lung disease (CLD) (*p* = 0.0004). With an AUC value of 0.766, the MSA is an adequate prognostic marker for the development of CLD. However, within the subgroup of right-sided hernias, no significant association between MSA and CLD disease was detectable (*p* = 0.3422). Nevertheless, the comparatively high AUC value of 0.929 should be addressed in further studies with a higher patient number.

In addition to the extent of pulmonary hypoplasia, cardiac impairment due to pulmonary hypertension and left ventricular hypoplasia is crucial for prognosis. According to Wang et al. (2022), the MSA value gives an indication of the extent of left heart hypoplasia [12]. Siebert et al. (1984) already classified left heart hypoplasia as a relevant risk factor in the pathogenesis of heart failure in patients with left-sided CDH; recent studies by Patel et al. (2019) and Prasad et al. (2022) came to a comparable conclusion [7,8,9]. According to a recent study showing that the mitigation of cardiac dysfunction leads to an improvement in the outcome of CDH, MSA should find its way into the prognostic criteria of right-sided hernias in the future [8].

We evaluated whether the MRI-based percentage area of the left ventricle to the total area of the ventricles (pALV) could serve as a quantitative parameter for cardiac stress and possibly enable a differentiated assessment of the severity of CDH and potential cardiovascular consequences. Regarding the prediction of the need for ECMO therapy, there was no significant association between pALV and this endpoint in the overall population (*p* = 0.0824). The pALV also did not significantly influence the development of CLD in the overall population (*p* = 0.2173). Concerning survival, the pALV showed no significant influence (*p* = 0.1134), indicating a comparable prediction of non-survival with chance. The lack of statistical significance of pALV as a prognostic parameter for the investigated outcome parameters could be related to the gestational age of the cohorts at the time of MRI examination (on average 30 + 5 weeks gestation). Massolo et al. (2019) demonstrated changes in cardiac dimension with gestational age, with no difference in LV area between healthy controls and CDH patients at 30–32 weeks [16]. Future studies should consider gestational age as an influencing factor on LV area as a risk factor for unfavorable outcomes in CDH.

To get to the bottom of why the investigated parameters MSA and pALV were not significantly associated with non-survival in the present cohort, the causes of death of the patients provide more precise information. A closer look at the causes of death in the right-sided hernia subgroup shows that in more than half (*n* = 6) of the patients, the diagnosis leading to death was severe untreatable pulmonary hypoplasia with consecutive massive limitations of ventilation and oxygenation. Accordingly, the o/e TFLV showed a significant correlation with survival/non-survival. Cerebral hemorrhage and fixed pulmonary hypertension were the cause of death in two other patients in right-sided hernia subgroup. This suggests that competing diagnoses not directly related to MSA or pALV led to death in most cases, which in turn may influence the non-significant correlation between survival/non-survival and pALV/MSA. 

Another limitation of our study is the non-cardiac cycle-gated acquisition of data. Therefore, cardiac movement and the cardiac cycle have strongly influenced our absolute measurements. 

Long-time fetal cardiac MRI (fCMR) was technically limited due to the movement of the fetus, the small size of the fetal structures, and the lack of real-time fetal electrocardiography (ECG) to synchronize the data with the fetal cardiac cycle [17]. 

Today, there are several technical options for fetal heart gating. Surface ECG is one of the preferred gating techniques in fetal cardiac MRI. The ECG records the different phases of the fetal cardiac cycle and synchronizes the imaging of the MRI with the different phases of the cardiac cycle [17]. The gating system ensures that the images of the fetal heart are taken exactly when it is in a certain phase of the cardiac cycle to minimize motion artifacts and obtain high-resolution images. During the scan, the pregnant woman lies on her back with the ECG electrodes placed on her skin. 

In addition to ECG gating, there are other methods of gating: metric-optimized gating and Doppler ultrasound gating (DUS-gating) [17,18]. With metric-optimized gating, data are recorded at an exaggerated time and a post-processing technique is used to assign the correct heart rate after scanning. 

MR-compatible DUS offers another option. The ultrasound device is based on Doppler waveforms of the heart rate trigger and is directly connected to the MR scanner [17,18]. However, this method is susceptible to movement. If the fetus moves out of the field of view, it must be repositioned. A third alternative is the so-called free-breathing fetal cine cardiac MRI based on DUS gating and tiny golden angle radial sampling (tyGRASP) with motion compensation [19].

In the future, ECG-gated acquisitions must be performed in order to overcome the limitations of our study and cardiac morphology should be reevaluated with regard to prognosis.

## 5. Conclusions

The MSA determined using fetal MRI is a good prognostic parameter for assessing ECMO requirement and CLD development in CDH and can possibly serve as an addition to established parameters. Its value in comparison to established parameters should be evaluated in future studies. Its value concerning right-sided hernias seems to be particularly promising and should be addressed in future studies with a larger patient cohort size.

## Data Availability

Research data are not shared due to data protection laws.

## Supplementary Materials

The following supporting information can be downloaded at: https://www.mdpi.com/article/10.3390/jcm13010268/s1, Table S1: Basic patient information.

## Appendix A

**Table A1:** Logistic regressionof MSA with respect to Non-Survival, ECMO-therapy, and CLD.

MSA	*p* (Non-Survival)	AUC (Non-Survival)	*p* (ECMO)	AUC (ECMO)	*p* (CLD)	AUC (CLD)
Total collective	0.4293	0.563	0.0054	0.678	0.0018	0.766
Left-sided hernia	0.5043	0.556	0.0285	0.652	0.0052	0.752
Right-sided hernia	0.5537	0.625	0.3380	0.938	0.3422	0.929

**Table A2:** Logistic regressionof pALV with respect to Non-Survival, ECMO-therapy, and CLD.

pALV	*p* (Non-Survival)	AUC (Non-Survival)	*p* (ECMO)	AUC (ECMO)	*p* (CLD)	AUC (CLD)
Total collective	0.1134	0.570	0.0824	0.597	0.2173	0.588
Left-sided hernia	0.2099	0.554	0.1378	0.594	0.2546	0.589
Right-sided hernia	0.2882	0.708	0.4255	0.688	0.5435	0.643

**Formula (A1)** Mathematical formula for the logistic regression of MSA-ECMO for the total collective.

(A1) $$R_{ECMO}=\frac{e^{-4.5682+\left(0.1071\right)\times MSA}}{1+e^{-4.5682+\left(0.1071\right)\times MSA}}$$

**Formula (A2)** Mathematical formula for the logistic regression of MSA-CLD for the total collective.

(A2) $$R_{CLD}=\frac{e^{-5.8752+\left(0.1628\right)\times MSA}}{1+e^{-5.8752+\left(0.1628\right)\times MSA}}$$
