# Supplementary material for: Fetal MRI-Based Mediastinal Shift Angle (MSA) and Percentage Area of Left Ventricle (pALV) as Prognostic Parameters for Congenital Diaphragmatic Hernia
Source: J Clin Med. 2024 Jan 3;13(1):268. doi: 10.3390/jcm13010268 (PMC10779621; doi:10.3390/jcm13010268)
Supplement: Supplementary file 1 [file jcm-13-00268-s001.zip › jcm-2764238-supplementary.pdf]

Table S1. Basic patient information.

Patient number	Gender (f=female, m=male)	Maturity at birth (GA)	GA MRI appointment	Side of hernia	Liver position	MSA	Volume right lung (ml)	Volume left lung (ml)	o/e FLV (%)	Mediastinal volume (cm3)	Thoracal volume (cm3)	FETO (1= no, 2=yes)	ECMO (ja=1, nein=2, contraindication=3)	ECMO duration (days)	Survival (1= no, 2= yes)	Days of life until death
1	m	36+4	29+5	rechts	up	left	1	13,1	26,2	9,5	111,82	1	1	10,04	1	10,3
2	f	39+0	28+0	rechts	up	left	1	7,3	18,3	9,09	64,03	1	1	8,83	2	
3	m	36+6	32+1	rechts	up	left	0,41	14,7	22,4	21,62	176,31	1	1	8,79	2	
4	f	39+1	24+6	rechts	down	left	9	11,3	62,8	13,98	61,99	1	2		2	
5	f	37+4	28+4	rechts	up	left	2,3	7,5	20,4	8,07	88,01	1	1	8	2	
6	f	37+0	29+3	rechts	up	left	7,7	16,3	45,8	16,56	68,44	1	2		2	
7	m	38+1	32+4	rechts	up	left	1,5	14,3	22,6	15,32	149,55	1	1	8	2	
8	m	37+3	32+2	rechts	up	left	8,1	15,6	34,7	30,24	197,94	1	1	13	1	28
9	f	39+2	30+3	links	up	left	1,3	13,8	26,2	13,85	109,34	1	1	9	2	
10	f	38+0	34+0	rechts	up	left	1,53	10,80	15,6	21,53	86,8	1	3		1	1
11	m	37+5	28+4	links	up	left	7,6	9,3	28,93	27,22	71,45	1	1	16	2	
12	m	38+4	37+0	links	up	right	20,8	2,1	22,7	21,03	169,81	1	2		2	
13	m	39+0	38+6	links	up	right	7,7	1,3	7,8	15,79	190,47	1	1	20	1	24
14	m	38+4	35+0	links	up	right	22,1	3,4	29,7	41,18	202,03	1	2		2	
15	m	38+0	25+6	links	up	right	11,8	2,8	40,3	7,91	62,53	1	1	6,83	1	34
16	f	37+6	31+6	links	down	right	33,4	24,4	88	21,59	122,86	1	2		2	
17	f	40+2	32+1	links	up	right	24,7	2,2	39,9	19,41	131,24	1	2		2	
18	f	38+1	31+2	links	up	right	16,4	1,8	29,2	13,23	94,31	1	2		2	
19	f	38+4	25+6	links	down	right	11,8	3	40,9	9,74	60,56	1	2		2	
20	m	37+4	31+1	links	up	right	17,9	1,7	31,8	15,88	101,91	1	1	5,42	2	
21	m	37+4	31+4	links	down	right	27,3	5,5	51,3	20,33	149,04	1	2		2	
22	m	35+4	31+4	links	up	right	9,8	0,1	15,1	19,02	153,58	1	1	8	2	
23	m	36+5	36+5	links	down	right	45	20	65,9	36,52	239,71	1	2		2	
24	m	39+3	32+1	links	down	right	29,2	19,1	71,6	29,71	114,55	1	2		2	
25	m	37+6	28+3	links	down	right	14,5	3,7	38,4	12,63	88,76	1	2		2	
26	f	38+2	24+2	links	down	right	8,5	1	31,4	8,66	46,22	1	2		2	
27	m	38+3	31+4	links	up	right	20,3	0	31,7	15,45	169,47	1	1	7,88	2	
28	f	36+0	33+6	links	up	right	17,2	0	22	22,88	178,75	2	3		1	0,25
29	f	37+2	36+3	links	up	right	38,9	8,8	49,46	41,08	192,03	1	2		2	
30	m	38+3	32+4	links	up	right	17,3	0,81	25,9	18,61	118,71	1	3		1	3
31	m	38+2	32+4	links	up	right	24,3	4,4	41	21,06	197,94	1	1	9	2	
32	m	38+0	33+1	links	up	right	22	7	51,7	9,45	106,96	1	1	7,4	2	
33	f	35+0	26+0	links	up	right	12,3	0,1	33,7	4,57	41,74	1	1	7,3	1	7
34	f	38+3	31+4	links	up	right	19,5	0	30,4	17,1	110,65	1	2		2	
35	f	38+3	30+0	links	down	right	21,8	0	39,4	17,99	124,45	1	2		2	
36	m	38+4	27+3	links	down	right	8,2	0	40,9	17,69	90,32	1	2		2	
37	f	37+1	30+5	links	up	right	11,9	0	20,1	25,5	141,07	1	1	11	2	
38	f	38+3	34+0	links	down	right	34,4	7,6	53,1	24,95	196,15	1	2		2	
39	m	38+5	28+0	links	up	right	13,2	11,5	54,4	10,48	44,88	1	2		2	
40	m	39+1	32+3	links	up	right	48,99	0	47,9	28,75	180,3	1	1	11,9	2	
41	m	38+6	29+1	links	down	right	21,7	7,5	57,3	12,05	107,41	1	2		2	
42	m	38+0	32+3	links	down	right	26,1	0	37,7	32,38	191,49	1	2		2	
43	f	38+4	28+3	links	up	right	24,1	6,7	59,6	10,78	73,31	1	2		2	
44	m	38+1	29+0	links	up	right	13,6	1,3	29,7	14	76,65	1	1	17,8	1	49,1
45	m	38+3	27+2	links	up	right	8,9	0	21,1	7,7	62,76	1	1	20	2	
46	f	38+0	28+2	links	down	right	33,4	8,2	88,9	15,77	93,92	1	2		2	
47	f	37+3	34+3	links	up	right	25,6	0	31,2	14,7	152,97	1	2		2	
48	m	38+4	29+0	links	down	right	16,7	0	33,3	17,97	96,7	1	2		2	
49	f	38+5	24+5	rechts	up	right	11,5	0	36,2	6,57	46,74	1	2		2	
50	f	39+0	28+7	rechts	down	right	23	1,1	48	18,99	137,38	1	1	7,1	2	
51	f	39+6	28+1	links	down	right	17,4	0	37,7	12,97	89,84	1	2		2	
52	m	38+2	28+5	links	up	right	11,7	0	24	10,82	83,83	1	1	5,9	2	
53	m	38+5	32+4	links	up	right	13	0	18,6	13,61	108,08	1	1	6,7	1	25,3
54	f	37+6	32+3	links	up	right	40,2	0	58,1	21,21	160,92	1	1	10,3	2	
55	f	37+1	27+4	links	up	right	9,1	0	20,9	10,75	75,29	1	1	10	1	10,2
56	f	38+2	34+0	links	down	right	26,9	2,3	36,4	26,21	149,73	1	2		2	
57	f	37+3	28+3	links	up	right	16,3	4,5	43,8	11,16	83,8	1	2		1	0,4
58	m	38+2	27+0	links	down	right	12,7	0	31	14,68	78,28	1	2		2	
59	m	36+0	31+6	links	down	right	21,6	0	32,9	11,89	118,2	1	1	kA	2	
60	f	38+1	28+3	links	up	right	14,6	0	30,8	9,47	84,45	1	1	8,5	2	
61	f	34+1	33+0	links	up	right	8,1	0	11	19,57	129,3	1	3		1	0,2
62	m	38+6	33+2	links	up	right	19	18	49,7	18,29	131,89	1	2		2	
63	m	39+0	33+2	links	down	right	36	3	52,3	22,09	141,52	1	2		2	
64	f	34+0	27+5	links	up	right	10,8	2	29	7,94	68,48	1	2		2	
65	m	35+2	31+0	links	up	right	18,1	0	29,8	14,09	88,11	1	2		2	
66	m	38+0	26+4	links	up	right	11	0	28	9,6	65,04	1	2		1	29
67	m	38+0	33+0	links	down	right	41,5	3,5	61,9	16,34	140,44	1	2		2	
68	f	37+4	33+4	links	up	right	31,06	0	40,7	24,11	180,64	1	1	10	2	
69	m	38+0	28+0	links	up	right	8,82	0	19,4	9,28	81,81	1	1	10	1	10
70	m	38+3	32+4	links	up	right	32,67	0	46,7	26,21	178,34	1	2		2	
71	m	38+3	31+3	links	up	right	7,38	0	11,7	21,73	94,9	1	1	4	1	6
72	m	37+4	31+3	links	up	right	24	0	38,6	12,63	114,21	1	2		2	
73	m	29+3	24+4	links	up	right	6,6	0	21	5,66	48,56	1	3		1	1
74	f	37+5	35+2	links	up	right	15,5	0	17,6	6,82	92,94	2	1	12	2	
75	f	35+4	29+4	links	up	right	12,3	0	23,3	7,1	52,12	1	3		1	8
76	m	37+4	33+1	links	down	right	37,3	9,8	78	19,6	130,23	1	2		2	
77	f	36+3	30+6	links	up	right	11,2	0	18,7	8,89	125,1	1	3		1	1
78	m	38+3	30+0	links	up	right	22	0	40	16,45	106,95	1	2		2	

79	f	37+6	34+2	links	up	right	16,3	1,7	22,2	23,05	149,73	1	1	9	2	
80	m	34+0	28+3	links	up	right	11,6	4,4	33,7	8,18	89,29	1	2		2	
81	f	38+4	34+5	links	down	right	25,01	0,1	29,89	21,26	147,21	1	1	9	1	9
82	f	38+1	36+1	links	up	right	23,8	4,1	29,6	16,16	156,87	1	2		2	
83	f	33+2	30+4	links	up	right	15,7	2	30,3	14,01	90,66	1	2		2	
84	m	38+0	29+1	links	up	right	10,7	2	24,9	10,32	82,43	1	2		2	
85	f	38+2	30+4	links	up	right	12,1	0,3	21,2	9,34	100,34	1	1	11	2	
86	m	38+2	33+4	links	up	right	0,5	11,5	15,7	16,01	100,02	2	3		1	1
87	m	38+2	36+1	links	down	right	27,4	7,1	37	26,18	153,58	1	2		2	
88	m	38+3	29+5	links	down	right	18,7	2,4	39,2	12,2	86,46	1	2		2	
89	m	35+2	31+2	links	up	right	17,9	2,3	32,4	19,34	118,06	1	1	8	1	31
90	f	38+0	35+6	links	up	right	16,9	0,6	19	17,83	114,29	1	1	11	1	30
91	m	36+2	29+1	links	up	right	9,6	1,4	21,6	17,33	81,6	1	3		1	2
92	m	40+1	23+0	links	down	right	7,1	0,9	30,9	5,81	30,15	1	2		2	
93	m	37+5	35+5	links	up	right	28	1,8	32,7	26,67	185,73	1	1	9	2	
94	m	38+6	32+0	links	up	right	18,9	3,4	33,5	20,76	91,15	1	2		2	
95	f	34+0	29+0	links	up	right	5,9	1	14,5	10,91	70,85	2	3		1	1
96	f	40+0	29+5	links	up	right	15,4	3,2	34,5	18,02	76,38	1	2		2	
97	m	36+6	29+3	links	up	right	10,1	0,4	20	9,52	110,67	1	2		2	
98	f	38+1	36+2	links	up	right	15,8	2,9	19,6	23,12	132,07	1	2		2	
99	m	38+6	26+0	links	up	right	14,8	4,1	48,7	14	71,64	1	2		2	
100	f	38+1	26+0	links	up	right	14,8	3,3	49,2	14,35	77,76	1	2		2	
101	m	38+1	32+1	links	up	right	13,9	2,1	24	12,44	92,07	1	1	15	1	15
102	f	36+6	27+6	links	down	right	12,1	1,7	30,82	16,53	89,73	1	1	2	1	3
103	f	38+3	27+4	links	down	right	15,5	1,7	39	11,22	69,19	1	2		2	
104	m	38+3	32	links	up	right	18,5	3,7	33,35	15,46	91,62	1	2		2	
105	f	38+2	29+1	links	up	right	12,3	1,9	27,87	10,97	91,07	1	1	4	2	
106	m	38+5	32+0	links	up	right	6,9	1,2	12,2	14,16	111,74	1	1	12	2	
107	f	38+1	28+4	links	down	right	16,9	5,3	46,1	8,9	53,15	1	3		1	1
108	f	37+2	34+0	links	up	right	17,5	3,5	25,59	22,38	145,98	1	1	8	2	
109	m	38+6	32+0	links	down	right	18,2	2	30,3	24,36	127,56	1	1	11	1	11
110	m	38+2	30	links	up	right	9,8	1,5	25,6	9,47	61,86	1	3		1	2
111	f	37+2	32+0	links	up	right	11,05	1,9	20,1	20,75	158,58	1	3		1	1
112	f	36+1	28+0	links	up	right	13,6	1,4	35	14,65	89,46	1	2		2	
113	f	38+5	34+1	links	up	right	28,83	6,3	43,85	26,16	205,36	1	2		2	
114	m	36+4	36+0	links	up	right	19,4	2,2	23,2	18	121,27	1	2		2	
115	m	38+3	28+0	links	down	right	12,19	2,6	32,08	17,87	93,85	1	2		2	
116	m	34+4	31+1	links	up	right	14,8	1,9	27,5	12,96	147,18	1	1	17	2	
117	f	37+1	30+3	links	down	right	19,3	4,4	41,1	23,5	98,5	1	2		2	
118	m	38+5	26+0	links	down	right	9,47	1,5	29,84	7,97	54,18	1	2		2	
119	m	38+0	25+0	links	up	right	7,7	2,78	31,89	10,62	43,49	1	2		2	
120	f	37+4	28+1	links	down	right	14,34	4,2	40,2	13,07	83,9	1	2		2	
121	m	37+2	29+3	links	up	right	9,93	0,6	20,1	15,4	91,33	1	1	10	2	
122	m	38+2	35+1	links	up	right	35,7	12,3	55,2	32,27	154,33	1	2		2	
